# Cold Induced Antisense Transcription of *FLOWERING LOCUS C* in Distant Grasses

**DOI:** 10.3389/fpls.2019.00072

**Published:** 2019-02-01

**Authors:** Fuchao Jiao, Kanchan Pahwa, Murray Manning, Niklas Dochy, Koen Geuten

**Affiliations:** Department of Biology, KU Leuven, Leuven, Belgium

**Keywords:** long non-coding RNA, lncRNA, *B. distachyon*, *in cis*, vernalization, *FLC*, positionally conserved, *COOLAIR*

## Abstract

Functional conservation of RNAs between different species is a key argument for their importance. While few long non-coding RNAs are conserved at the sequence level, many long non-coding RNAs have been identified that only share a position relative to other genes. It remains largely unknown whether and how these lncRNAs are conserved beyond their position. In *Arabidopsis thaliana*, the lncRNA *COOLAIR* is transcribed antisense from *FLOWERING LOCUS C* (*FLC*) in response to cold. Despite relatively low sequence similarity, the *COOLAIR* expression pattern and *in vitro* RNA secondary structure are highly conserved across the family Brassicaceae, which originated some 50 mya. It is unclear, however, whether *COOLAIR* functions in distantly related species such as monocots, which diverged some 150 mya. Here, we identified antisense lncRNAs from *FLC* homologs in various monocot species that share no sequence similarity with *A. thaliana COOLAIR*. Yet similar to *COOLAIR*, we found that *BdODDSOC1* antisense (*BdCOOLAIR1*) and *BdODDSOC2* antisense (*BdCOOLAIR2*) are induced by cold in a *Brachypodium distachyon* winter accession. Across *B. distachyon* accessions, the sequences of *BdCOOLAIR1* and *BdCOOLAIR2* are less conserved than exons but more conserved than flanking regions, suggesting a function for the transcript itself. Knock down of the *BdODDSOC2* non-overlapping *BdCOOLAIR2* transcript did not show a morphological phenotype, but did result in significantly higher *BdODDSOC2* expression during cold, indicating that *BdCOOLAIR2* performs a role *in cis* in the rate of *BdODDSOC2* silencing. This functional similarity between eudicot and monocot species reveals ancient conservation or convergent evolution of *FLC* antisense transcription. Either scenario supports its functional importance.

## Introduction

Thousands of long non-coding RNAs (lncRNAs) have been identified and new lncRNAs are continuously being discovered. Yet, the functional importance of these transcripts remains debated. One reason is that lncRNAs evolve much faster than protein coding genes, which has been observed frequently by comparing genome and transcriptome sequences between various species (Necsulea et al., 2014; Hezroni et al., 2015). While sharing low primary sequence conservation, a few better studied lncRNAs, such as *XIST* (Yildirim et al., 2013), *HOTAIR* (Somarowthu et al., 2015), *TUNA* (Lin et al., 2014) and *cyrano* (Ulitsky et al., 2011), have been experimentally demonstrated to have conserved secondary structures or functions between species. Across different plant species, only hundreds of lncRNAs are conserved at the sequence level, while many more are only positionally conserved (Mohammadin et al., 2015; Wang et al., 2015). To date, little has been done to explore the function of these positionally conserved lncRNAs.

*COOLAIR* is a set of lncRNAs transcribed antisense from *FLOWERING LOCUS C* (*FLC*) in *Arabidopsis thaliana* (Swiezewski et al., 2009). Despite the relatively low nucleotide sequence identity, *COOLAIR* shows high expression and secondary structure conservation across the Brassicaceae (Castaings et al., 2014; Hawkes et al., 2016; Li et al., 2016). *FLC* is a MADS box gene and one of the most intensively studied plant genes. It is best known for its function as a repressor of flowering (Michaels and Amasino, 1999; Bloomer and Dean, 2017; Whittaker and Dean, 2017). To allow flowering in spring, *FLC* needs to be repressed by the autonomous pathway and the vernalization pathway. In the autonomous pathway, *FLC* is promoted by *FRIGIDA* (*FRI*), while repressed by FCA. In the vernalization pathway, *FLC* expression is reduced by prolonged cold and the locus is epigenetically silenced. *COOLAIR* is involved in both of these pathways. In the autonomous pathway, FCA, FY, CstF64, and CstF77 promote the use of the proximal splice site of *COOLAIR* to represses *FLC* through an FLD-dependent reduction in H3K4me2 (Liu et al., 2010). In response to vernalization, *COOLAIR* is transiently induced by prolonged cold, reaching a maximum expression level after 2 weeks (Swiezewski et al., 2009). Although *COOLAIR* is not absolutely required for the silencing of *FLC* during laboratory vernalization (Helliwell et al., 2011; Csorba et al., 2014), *FLC* terminator/*COOLAIR* promoter exchange lines result in the slowing down of the *FLC* silencing rate, through the switch in chromatin state by erasing H3K36me3 (Csorba et al., 2014). In the slowly vernalizing accession Var2-6, *FLC* repression is slower due to splicing of the distally polyadenylated *COOLAIR* (Li et al., 2015), indicating the role of *COOLAIR* in natural variation. The stable silencing of *FLC* is associated with two more lncRNAs, *COLDWRAP* and *COLDAIR*, which recruit PHD-PRC2 to a specific chromatin region (Heo and Sung, 2011; Kim and Sung, 2017; Kim et al., 2017). However, *COOLAIR* has not been identified beyond the Brassicaceae, both *COLDWRAP* and *COLDAIR* have not even been found beyond *A. thaliana* (Castaings et al., 2014; Li et al., 2016).

*FLOWERING LOCUS C* has been identified in monocots through synteny and phylogenetic analysis (Ruelens et al., 2013). *FLC* homologs in barley, wheat and *Brachypodium distachyon*, a model for the temperate cereals, are repressed during vernalization, showing a similar expression behavior as *FLC* in *A. thaliana* (Greenup et al., 2010; Sharma et al., 2017). In *B. distachyon*, three *FLC* homologs have been found: *BdODDSOC2*, *BdODDSOC1*, and *BdMADS37* (Ruelens et al., 2013). *BdODDSOC2* is epigenetically silenced during vernalization in the winter accession BdTR3C and overexpression of *BdODDSOC2* in the facultative accession Bd21-3 delays flowering time (Sharma et al., 2017). Besides temperate monocots, there are *FLC* homologs in subtropical monocots, such as *OsMADS51* in *Oryza sativa*, which is proposed to be involved in promoting flowering in short days (Kim et al., 2007; Ruelens et al., 2013). In contrast to the extensively studied mechanisms of *FLC* in *A. thaliana*, whether and how lncRNAs function in the regulation of *FLC* homologs in distantly related monocots is unknown.

Here we show that the antisense transcript *COOLAIR* is positionally conserved in *FLC* homologs across distantly related monocot species. We found that in *B. distachyon* accession BdTR3C, *BdCOOLAIR2* is strongly induced by prolonged cold and represses its neighboring coding gene *BdODDSOC2*. The functional similarity of *FLC*/*COOLAIR* sense-antisense pairs between eudicots and monocots highlights the functional importance of antisense long non-coding RNA transcription.

## Materials and Methods

### Plant Growth Conditions

*Brachypodium distachyon* seeds were overnight incubated on wet filter paper in petri dishes, then sown in root trainers (soil:vermiculite = 2:1). For qPCR, root trainers were putted in 8 h/16 h light/dark at 23°C for 3 weeks, then 8 h/16 h light/dark at 4°C for 2 weeks, and finally transferred to 8 h/16 h light/dark at 23°C for 1 week. For phenotyping, root trainers were placed in 8 h/16 h light/dark at 23°C for 3 weeks, then transferred to 8 h/16 h light/dark at 4°C for 2 weeks (Bd21-3) and 6 weeks (BdTR3C), respectively, then transferred to 20h/4h light/dark at 23–25°C until flowering.

### RNA Extraction, cDNA Synthesis, and qPCR Analysis

RNA extraction was performed following standard TRIsure protocol. DNA was removed using TURBO DNA free kit (Ambion-Applied), first single strand cDNA was synthesized by SensiFast cDNA Synthesis Kit of Bioline (GC Biotech BIO-65054). qPCR was performed with SensiFast SYBR Hi-Rox Kit (GC Biotech: BIO-92020), at least three biological replicates and two technical replicates were used for each time point.

### Genomic Sequence Diversity

*Brachypodium distachyon* pan*-*genome sequences were downloaded from the COGE database. Alignment was performed by CLC sequence viewer 7.7, with gap open cost 10.0, gap extension cost 1.0. DNA polymorphisms were calculated by DNASP, sites with alignment gaps were excluded. The figure was drawn with a 50-sites sliding window and 25 sites step size.

### RNAi Lines

For RNAi mediated knockdown of *BdCOOLAIR2*, around 200 bp of *BdCOOLAIR2* was first cloned into a pENTR2B vector (Invitrogen), with BamHI (Promega) and EcoRV (Promega) restriction enzymes. Then, the *BdCOOLAR2* sequence was inserted into a binary destination vector pIPK007 with the *Zea mays* UBIQUITIN promoter (Himmelbach et al., 2007) by using Gateway^TM^ LR Clonase^TM^ Enzyme mix (Invitrogen). The primers for *BdCOOLAIR2* cloning are as follows: *BdCOOLAIR2*_RNAi_F: TGGGTC GGATCCGTCCGGAGGCACACAAAT and *BdCOOLAIR2*_RNAi_R: CAAACTGATATCGGGACCTGAAGAACACGAGA. *B. distachyon* transformation was performed according to the *Agrobacterium*-mediated transformation protocol (Himmelbach et al., 2007; Alves et al., 2009). The primers for genotyping are listed in Supplemental Table 1. Phenotyping was done with T1 plants in a growth chamber, null sibling plants were used as controls (NC).

### Calculation of Maximal Information Coefficient

We made use of the Minerva package in R to calculate the MIC of the data (Albanese et al., 2013). To test for significance, we generated 250000 permuted datasets and calculated the MIC for each of these and calculated a *p*-value as the fraction of datasets with an MIC higher than the MIC for the observed data.

## Results

### *FLC* Homologs in *B. distachyon* and Other Grasses Generate Antisense LncRNAs

**FIGURE 1:**
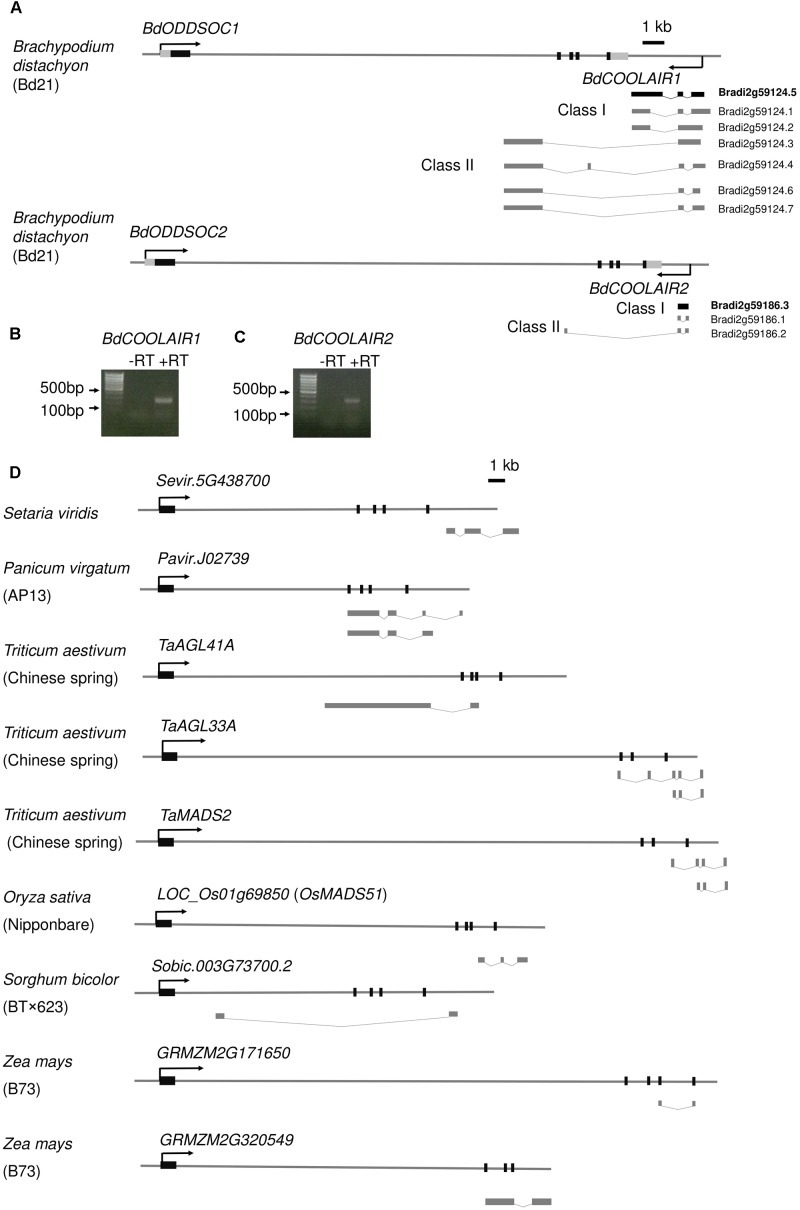
*FLC* homologs in *B. distachyon* and other monocots generate antisense long non-coding RNAs. **(A)** Schematic representation of annotated *FLC* homolog genomic loci (*BdODDSOC1* and *BdODDSOC2*) and antisense transcripts in *B. distachyon*. For *BdODDSOC1* and *BdODDSOC2* genomic loci, the black boxes indicate exons, the black lines indicate introns and flanking region. The directions of sense transcripts (black arrow), Class I antisense transcripts and Class II antisense transcripts are shown. Transcripts in bold (Bradi2g59124.5 and Bradi2g59186.3) are confirmed by RT-PCR. **(B)** and **(C)** Agarose gel for *BdCOOLAIR1* and *BdCOOLAIR2*, respectively after RT-PCR. A DNA ladder is shown. **(D)** Schematic representation of *FLC* homolog genomic loci and antisense transcripts in various grasses. For *FLC* homologs, the black boxes indicate exons, the black lines indicate introns and flanking regions. The direction of sense transcripts (black arrow) is shown.

To investigate whether there are non-coding RNAs generated from *FLC* loci in monocots, we first analyzed publicly available datasets. In *B. distachyon*, we found multiple Expressed Sequence Tags (ESTs) behind the stop codon of *BdODDSOC1* and *BdODDSOC2*, based on the RNA-seq and EST data in the phytozome browser (Goodstein et al., 2012) (Figure 1A). The direction of these ESTs is antisense to *BdODDSOC1* and *BdODDSOC2* coding transcripts. They are annotated as lncRNAs in GreeNC, a database of plant lncRNAs (Goodstein et al., 2012; Paytuví Gallart et al., 2015). For *BdODDSOC1*, there are 7 annotated antisense ESTs, while only one (Bradi2g59124.5) has high confidence with a full-length of 2581 bp. For *BdODDSOC2*, there are 2 annotated antisense ESTs, both with high confidence. The length of those is 475 bp (Bradi2g59186.1) and 372 bp (Bradi2g59186.2), respectively. The coding potential for these three antisense transcripts is very low, –1.130 (Bradi2g59124.5), -1.034 (Bradi2g59186.1) and –1.023 (Bradi2g59186.2), further suggesting that they could function as non-coding transcripts.

We further confirmed the existence of these antisense transcripts by RT-PCR and Sanger sequencing. Our results are not fully consistent with the EST data, because we could only amplify the high-confidence proximal antisense of *BdODDSOC1* and *BdODDSOC2* (Figure 1B,C). The RT-PCR confirmed that the antisense transcripts are not overlapping with *BdODDSOC1* and *BdODDSOC2* (Supplementary Figure S1), which is different from *A. thaliana* (Swiezewski et al., 2009). Class II lncRNAs are also annotated from EST data while we could not amplify Class II *BdCOOLAIR2*. We designed primers specific for Class II, but amplified nothing. For Class I *BdCOOLAIR2*, we got two bands, the bands of Bradi2g59186.3 is much stronger than Bradi2g59186.1 (Figure 1A). Both Bradi2g59186.3 and Bradi2g59186.1 are Class I *BdCOOLAIR2*. Even though we could not amplify distal antisense transcripts, we cannot exclude their existence because they could be expressed in other accessions or under specific conditions which have not been tested in our study.

To know whether *FLC* antisense transcripts are also present in other monocots, we further analyzed the public gene expression data available for other grass species. Interestingly, we found that *FLC* homologs in at least 6 other grasses produce antisense transcripts, including the model plant *Setaria viridis*, the energy plant *Panicum virgatum*, the temperate cereal *Triticum aestivum* and the tropical cereals *Oryza sativa*, *Sorghum bicolor* and *Zea mays* (Figure 1D). Thus, we concluded that *FLC* antisense transcription is present in various grass species.

To know whether these antisense transcripts are *COOLAIR* homologs, we analyzed their sequence conservation. The sequence has fully diverged and no sequence conservation could be detected between these antisense transcripts and *COOLAIR* in *A. thaliana* (Supplementary Figure S2). Even between grass species, the antisense transcripts share no sequence conservation (Supplementary Figure S3). Because they are all located at the end of *FLC* homologs, we propose that these antisense transcripts are positionally conserved *COOLAIR* homologs in grasses. For further study, we named the antisense of *BdODDSOC1* and *BdODDSOC2* as *BdCOOLAIR1* and *BdCOOLAIR2*, respectively.

### *ODDSOC2* Antisense Transcription Is Strongly Induced by Cold in a *B. distachyon* Winter Accession

**FIGURE 2:**
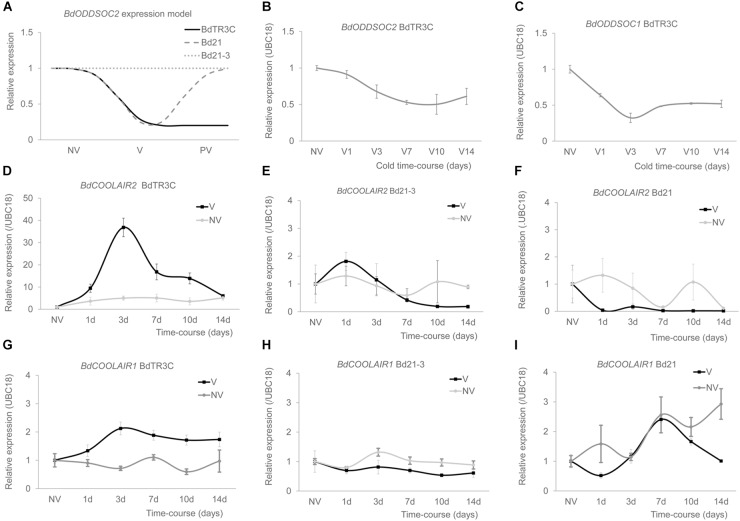
*BdCOOLAIR2* and *BdCOOLAIR1* are upregulated by vernalization. **(A)** Model representation of expression patterns of *BdODDSOC2* in three accessions: BdTR3C, Bd21 and Bd21-3. NV is non-vernalized, V is vernalized, PV is post-vernalized. In BdTR3C, *BdODDSOC2* is epigenetically silenced in vernalization (V) and stays down in post-vernalization (PV); in Bd21, *BdODDSOC2* is repressed in vernalization, while it comes back up post-vernalization; in Bd21-3, *BdODDSOC2* expression is not affected during vernalization and post-vernalization. **(B)** *BdODDSOC2* is repressed during vernalization. **(C)** *BdODDSOC1* is repressed during vernalization. **(D)** *BdCOOLAIR2* in BdTR3C is induced by vernalization (black). Non-vernalization control is shown (gray). **(E)** *BdCOOLAIR2* expression in Bd21-3, vernalization (black) and non-vernalization (gray) are shown. **(F)** *BdCOOLAIR2* expression in Bd21, vernalization (black) and non-vernalization (gray) are shown. **(G)** *BdCOOLAIR1* in BdTR3C is induced by vernalization (black). Non-vernalization control is shown (gray). **(H)** *BdCOOLAIR1* expression in Bd21-3, vernalization (black) and non-vernalization (gray) are shown. **(I)** *BdCOOLAIR1* expression in Bd21, vernalization (black) and non-vernalization (gray) are shown. Values are means ± SEM of three or four biological replicates, two technical replicates.

To investigate the expression patterns of these positionally conserved lncRNAs, we performed quantitative RT-PCR in three accessions. We have previously shown that in the winter accession BdTR3C, *BdODDSOC2* is epigenetically silenced by prolonged cold (Figure 2A), while in the spring accession Bd21, *BdODDSOC2* is repressed by cold but not stably silenced. In the facultative accession Bd21-3, *BdODDSOC2* does not respond to cold (Sharma et al., 2017). Interestingly, we found that in BdTR3C, *BdODDSOC2* and *BdCOOLAIR2* show opposite expression patterns during early stage cold treatment (Figure 2B,D and Supplementary Figure S4). *BdODDSOC2* goes down, while *BdCOOLAIR2* is significantly upregulated (approximately 40 folds) and reaches a maximal level after 3 days of cold. To investigate whether *BdCOOLAIR2* is specifically induced by cold, we compared its expression to non-cold treatment control conditions. Indeed, without cold, *BdODDSOC2* antisense expression is not induced (Figure 2D). To verify whether the relationship between *BdCOOLAIR2* and *BdODDSOC2* expression holds in a facultative or a spring accession, we checked the expression of *BdCOOLAIR2* in Bd21-3 and Bd21. *BdCOOLAIR2* is only slightly induced in Bd21-3 (approximately two folds) after 1 day (Figure 2E), and *BdCOOLAIR2* is not induced significantly in Bd21 (Figure 2F).

For *BdODDSOC1*, *BdCOOLAIR1* also shows an opposite expression pattern in BdTR3C (Figure 2C,G). *BdODDSOC1* goes down, while *BdCOOLAIR1* goes up, again reaching a maximum level after 3 days of cold. Here only an approximately two-fold induction level is reached. Similar to *BdCOOLAIR2*, *BdCOOLAIR1* is induced by cold (Figure 2G). Similar to *BdCOOLAIR2*, *BdCOOLAIR1* is not induced in Bd21-3 (Figure 2H) or Bd21 (Figure 2I).

### *BdODDSOC1* and *BdODDSOC2* Intronic Transcripts Are Not Induced by Prolonged Cold

Similar to the *A. thaliana FLC* locus, the *BdODDSOC1* and *BdODDSOC2* loci have a very large first intron, suggesting that this may play a role in gene regulation. To investigate whether intronic sense lncRNA transcription, similar to *COLDAIR*, is present in *B. distachyon*, we investigated RNA-seq data as no ESTs were annotated for the introns. We found that there are multiple peaks located in the long first intron of *BdODDSOC1* and *BdODDSOC2* and considered that these peaks might point to intronic long non-coding RNAs in these regions. To verify this idea in BdTR3C, we designed primers spanning the entire first intron of *BdODDSOC1* and *BdODDSOC2* (Supplementary Tables S2, S3). After RT-PCR amplification, we observed a series of bands along the first intron of *BdODDSOC1* and *BdODDSOC2* (Supplementary Figures S5, S6). However, this did not generate a clear idea about the number of different lncRNAs transcribed from the first intron, nor of the size of possible lncRNAs.

To know whether the transcripts observed in the first intron are induced by prolonged cold, we performed quantitative RT-PCR. They showed a similar expression pattern as sense coding transcripts, and are downregulated during prolonged cold (Supplementary Figures S7, S8). But because long non-coding RNAs may be only induced at a specific time and under specific conditions, which may have gone unnoticed in our experiments, we cannot conclude whether they function similar to *COLDAIR*.

### The *BdCOOLAIR* Sequence Is Relatively Conserved in *B. distachyon*

**FIGURE 3:**
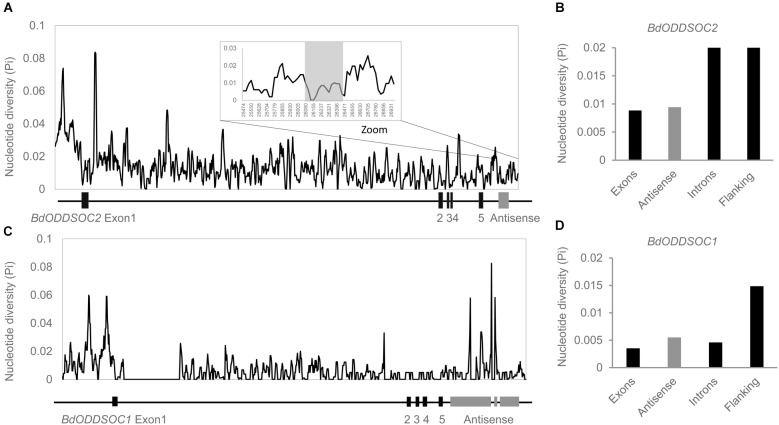
*BdCOOLAIR2* sequence is more conserved than intronic and flanking regions. **(A)** Sliding window plot representation of *BdODDSOC2* genomic diversity, with a window size of 28 kb. **(B)** Bar chart of sequence diversity of different regions of *BdODDSOC2*. **(C)** Sliding window plot representation of *BdODDSOC1* genomic diversity, with a window size of 23 kb. **(D)** Bar chart of sequence diversity of different regions of *BdODDSOC1*. Black indicates exons, introns and flanking regions, gray indicates antisense.

To know how the antisense sequence evolved, we analyzed the DNA sequences using the released *B. distachyon* pan-genome sequencing data (Gordon et al., 2017). We downloaded the genomic DNA sequence of *BdODDSOC2* and *BdODDSOC1* for 54 of the genome sequenced *B. distachyon* accessions and conducted a polymorphism analysis (Figure 3).

*BdODDSOC2* contains five exons, which are most conserved with a nucleotide diversity value (NDV) of 0.008. The intronic and gene flanking region NDV is 0.0209 and 0.0217, respectively. The antisense value (0.009) is higher than the value for exons, but lower than introns and flanking regions. Thus, in *BdODDSOC2*, the antisense region evolved slightly faster than exons but much more slowly than regions under more neutral selection (introns and flanking regions). This suggests a function for the RNA transcript and not just for the act of transcription (Kopp and Mendell, 2018). *BdODDSOC1* shows a comparable result, where the antisense sequence (NDV is 0.005) evolved faster than exons (NDV is 0.0035) and introns (NDV is 0.0045), but more slowly than flanking regions (NDV is 0.015). Therefore, we wanted to test whether *BdCOOLAIR2* performs a function at the transcript level rather than testing whether the action of transcription is important.

### Silencing of *BdODDSOC2* Is Delayed in *BdCOOLAIR2* Knock-Down Lines

We have shown that the position of *BdCOOLAIR2* does not overlap with *BdODDSOC2* (Figure 1A and Supplementary Figure S1), and its sequence evolution suggests that *BdCOOLAIR2* has a function at the RNA level (Figure 3A,B). To understand the functional importance of *BdCOOLAIR2*, we used RNA interference to knock down *BdCOOLAIR2* in two accessions: BdTR3C and Bd21-3. We chose *BdCOOLAIR2* rather than *BdCOOLAIR1*, because in BdTR3C, *BdODDSOC2* is epigenetically silenced by cold and *BdCOOLAIR2* is much more strongly induced than *BdCOOLAIR1*. We generated 10 RNAi lines for accession BdTR3C and 18 lines for accession Bd21-3.

**FIGURE 4:**
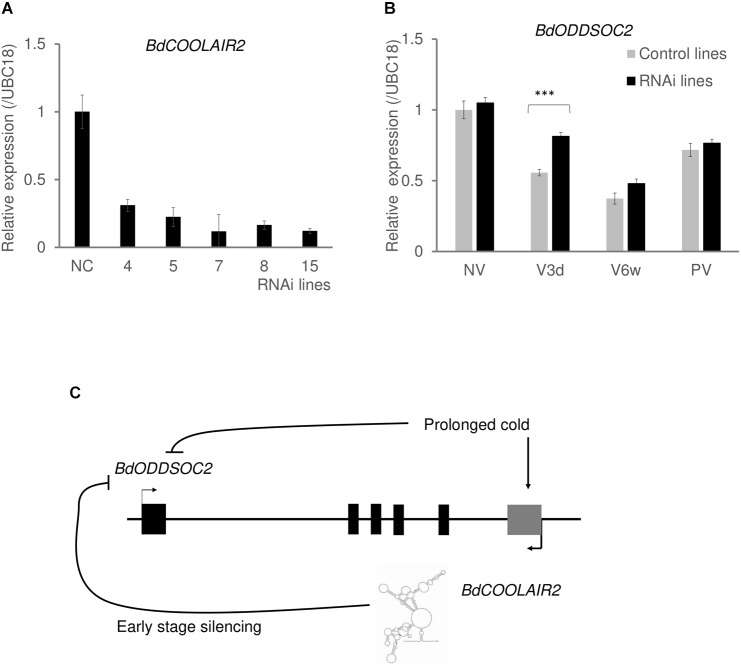
*BdCOOLAIR2* function in the silencing of *BdODDSOC2*. **(A)** Expression of *BdCOOLAIR2* in BdTR3C RNAi lines is significantly lower than in null sibling control lines (NC). Values are means ± SEM of three biological replicates. **(B)** Silencing of *BdODDSOC2* is delayed in *BdCOOLAIR2* knock-down lines. *BdODDSOC2* expression was tested in *BdCOOLAIR2* RNAi lines, in non-vernalization (NV), three days of vernalization (V3d), 6 weeks of vernalization (V6w) and 1 week post-vernalization (PV). Gray bars indicate null-sibling control lines, black bars indicate RNAi lines. Values for control lines are means ± SEM of three biological replicates, while values for RNAi lines are means ± SEM of twelve to fifteen biological replicates, pooled of all RNAi lines. The values of each RNAi line were separately shown in Supplementary Figure S9. Asterisks indicate statistically significant differences with the student’s *t*-test (^∗∗∗^*p* < 0.001). **(C)** In response to vernalization, the *BdCOOLAIR2* transcript has a function associated with silencing of *BdODDSOC2*.

To investigate whether we successfully knocked down *BdCOOLAIR2* using RNAi, we first checked its expression level in RNAi T0 and T1 lines. We confirmed that in induced conditions, *BdCOOLAIR2* expression is lower in 5 RNAi lines compared to null sibling controls (Figure 4A and Supplementary Table S4). To study whether *BdCOOLAIR2* works *in cis*, we investigated the expression level of its neighboring coding transcript *BdODDSOC2*. Interestingly, in BdTR3C, there is a significant difference in *BdODDSOC2* expression level between RNAi and null siblings at three days of cold treatment (*t*(16) = 4.3258, *p* < 0.001, Figure 4B, Supplementary Figure S9 and Supplementary Tables S5, S6). However, in non-vernalization, 6 weeks of vernalization and post-vernalization conditions, we did not observe a significant difference. Three days of cold treatment is the time point when *BdCOOLAIR2* reached its maximum expression level, supporting a role for *BdCOOLAIR2* in regulating *BdODDSOC2* during cold treatment. Although *BdODDSOC2* is still repressed in these RNAi lines, the silencing rate is slower compared with control lines (Figure 4B and Supplementary Figure S11A). *BdODDSOC2* and *BdODDSOC1* share a lot of sequence similarity. To know whether *BdODDSOC1* can be induced in *BdCOOLAIR2* RNAi lines, we also tested the expression of *BdODDSOC1.* We found that after three days of cold treatment, *BdODDSOC1* is not induced in *BdCOOLAIR2* RNAi lines, which is different with *BdODDSOC2* (Supplementary Figure S10). We conclude that like *COOLAIR* in *A. thaliana*, *BdCOOLAIR2* has a function associated with the rate of *BdODDSOC2* silencing in a winter accession (Figure 4C and Supplementary Figure S11B) (Csorba et al., 2014). We showed previously that ectopic expression of *BdODDSOC2* delays flowering in Bd21-3. As a result, we would expect an effect of silencing of *BdCOOLAIR2* on flowering time because *BdODDSOC2* is silenced more slowly. We used 10 BdTR3C RNAi lines and 11 Bd21-3 RNAi lines to phenotype flowering time and leaf number. However, we did not observe a significant difference between these lines and controls (Supplementary Figure S12). This suggests that the effect on expression of *BdODDSOC2* is not strong enough to result in a significant phenotype.

To further test the antagonistic relationship between *BdODDSOC2* and *BdCOOLAIR2* (Rosa et al., 2016), we investigated their gene expression levels using qRT-PCR in 74 three-week old individual plants belonging to different accessions (Supplementary Table S7). We observed that in plants with low *BdCOOLAIR2* expression, *BdODDSOC2* expression levels can be high and in plants with high *BdCOOLAIR2* expression, *BdODDSOC2* expression levels are almost always low (Supplementary Figure S13). This suggests a non-coexistence relationship, in which either transcript can be present, but they are not expressed together. To formally test such a non-linear association, we made use of the maximal information coefficient (MIC), which measures linear or non-linear correlation between paired variables (Reshef et al., 2011). We found that the calculated MIC for the data (MIC = 0.3752117) is significantly higher than the MICs calculated for a null distribution of permuted datasets of equal size (empirical *p* value = 0.0127). This further confirms the antagonistic relationship between *BdCOOLAIR2* and *BdODDSOC2.*

## Discussion

The expression of *COOLAIR* and its structure is conserved across closely related Brassicaceae (Castaings et al., 2014; Hawkes et al., 2016; Li et al., 2016). In our study, we demonstrate that beyond the Brassicaceae, *COOLAIR* is positionally conserved in distantly related monocot species. However, its sequence is unrecognizable. In *B. distachyon*, we find that *BdCOOLAIR2* is strongly induced by prolonged cold in a vernalization requiring accession but not in spring accessions. We further demonstrate that *BdCOOLAIR2* sequence diversity is lower than intronic and gene flanking regions, suggesting a function for the transcript itself. We indeed found that the *BdCOOLAIR2* transcript plays a role in the repression of *BdODDSOC2*.

Sense-antisense pairs are pervasive in bacteria, budding yeast, rice, maize and mammalian genomes (Pelechano and Steinmetz, 2013). LncRNA sequences evolve rapidly, and antisense lncRNAs are more positionally conserved than sequence conserved (Necsulea et al., 2014; Hezroni et al., 2015; Mohammadin et al., 2015; Wang et al., 2015). However, only a few studies have been performed to compare the function of lncRNAs across distantly related species (Ulitsky et al., 2011; Yildirim et al., 2013; Lin et al., 2014; Somarowthu et al., 2015). Our data provide more evidence that positionally conserved antisense long non-coding RNAs can be deeply functionally conserved or alternatively, can originate through convergent evolution to fulfill a similar role in gene regulation. The first hypothesis appears more likely as the lack of sequence similarity can be a consequence of sampling density and not enough sequence data is available to fully explore the rapid sequence evolution of lncRNAs over long evolutionary distances. Indeed, *FLC* homologs in intermediate species such as *Theobroma cacao* and *Vitis vinifera* also have annotated antisense transcripts (Supplementary Figure S14).

In *A. thaliana*, alternative polyadenylation is important for *COOLAIR* function (Liu et al., 2010; Li et al., 2015) and proximal *COOLAIR* represses *FLC* while distal *COOLAIR* is not involved in repression. In *B. distachyon*, we only amplified proximal antisense lncRNAs, which is consistent with *Arabis alpina*, in which proximal antisense is much more conserved than distal antisense (Castaings et al., 2014). However, in *Brassica rapa*, it was shown that the distal antisense is strongly induced by cold (Li et al., 2016). Therefore, it remains somewhat unclear whether alternative polyadenylation is a conserved aspect of *COOLAIR* function. In contrast to the antagonistic relationship in a single cell between *FLC* and *COOLAIR* (Rosa et al., 2016), it has been recently reported that FRI-containing supercomplex and FRI partners upregulate both *FLC* and Class II *COOLAIR*, by promoting the formation of a chromatin loop around *FLC* locus (Li et al., 2018). In addition, elimination of Class II RNAs in the FLC+MAF2-T and FLC+NOS-T lines is associated with a reduction in *FLC* expression. Thus, the mechanism behind *FLC*/*COOLAIR* antagonistic expression and *COOLAIR* guided *FLC* silencing under different pathways needs to be further studied.

We tried to identify sense intronic lncRNAs, but we did not reach a conclusion. The VRE region (promoter of *COLDAIR*) has evolved rapidly in Brassicaceae (Castaings et al., 2014; Li et al., 2016), with very low sequence conservation. Thus, so far *COLDAIR* has not been found beyond *A. thaliana*. We found some transcripts based on RNA-Seq, EST and our RT-PCR data. These transcripts show a similar expression pattern as sense coding transcripts, and they are downregulated during prolonged cold. We propose that these transcripts might be unspliced sense *FLC* transcripts. However, we cannot exclude the existence of non-coding transcripts in the introns. The reason might be that we did not use suitable accessions or conditions that would induce lncRNAs expression.

In *A. thaliana*, the sequence of *COOLAIR* overlaps with *FLC*, which makes the use of RNAi limited to understand its function. Therefore, *FLC* terminator/*COOLAIR* promotor exchange lines were created to truncate the expression of *COOLAIR* (Wang et al., 2014). In the case of *B. distachyon*, *BdCOOLAIR2* does not overlap with *BdODDSOC2* and we generated RNAi lines specifically targeting the antisense lncRNA. In no-cold and post-cold conditions, we did not see a clear effect on *BdODDSOC2*, however at 3 days of cold, *BdODDSOC2* expression is higher in antisense knockdown lines compared with controls. Thus, we demonstrated that *BdCOOLAIR2* functions *in cis*, and at the RNA level. *BdCOOLAIR2* knock down does not affect the overall expression of *BdODDSOC2* but slows down the repression process. This effect apparently was not strong enough to affect flowering time. However, considering the slow repression of *FLC* caused by splicing of the distally polyadenylated *COOLAIR* in the slowly vernalizing accession Var2-6 (Li et al., 2015), it has been proposed that *COOLAIR* works more importantly in natural conditions in which temperature and other factors fluctuate widely (Whittaker and Dean, 2017). It would be very interesting to verify this hypothesis also in *B. distachyon*.

## Author Contributions

KG and FJ designed the experiments. FJ, KP, and MM carried out the experiments and analyzed the data. ND designed the primers for *BdODDSOC1* and *BdODDSOC2* introns. FJ and KG wrote the manuscript.

## Conflict of Interest Statement

The authors declare that the research was conducted in the absence of any commercial or financial relationships that could be construed as a potential conflict of interest.
