# Supplementary material for: Cold Induced Antisense Transcription of FLOWERING LOCUS C in Distant Grasses
Source: Front Plant Sci. 2019 Feb 1;10:72. doi: 10.3389/fpls.2019.00072 (PMC6367677; doi:10.3389/fpls.2019.00072)
Supplement: Supplementary file 8 [file Data_Sheet_1.pdf]

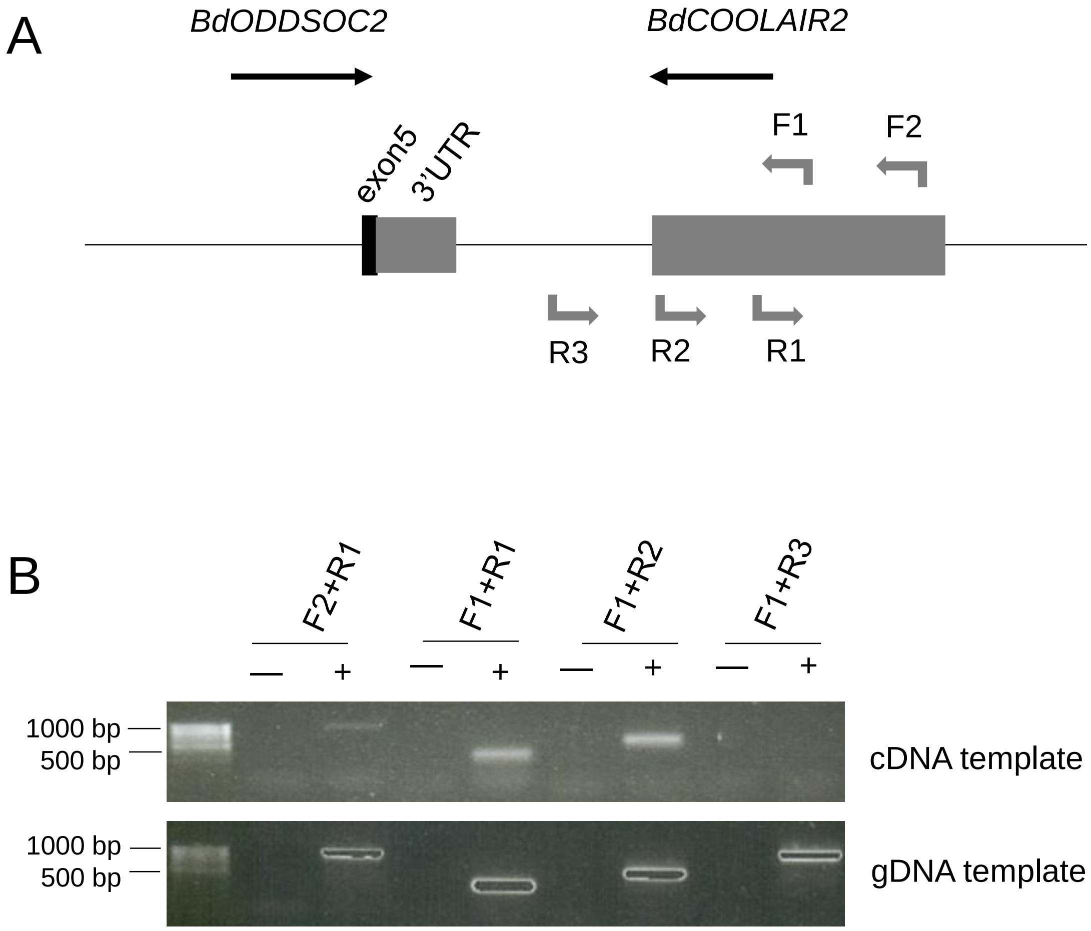

FIGURE S1. *BdCOOLAIR2* does not overlap with *BdODDSOC2*. (A) Genomic position of *BdODDSOC2* and *BdCOOLAIR2*. Black box indicates the fifth exon of *BdODDSOC2*. Gray box indicates *BdCOOLAIR2*. Black arrows indicate transcription direction. Primers are shown. **(B)** RT-PCR for different primers pairs around *BdCOOLAIR2*. Using primer F1 and R3 can not amplify anything indicating that the right border of *BdOOLAIR2* is away from *BdODDSOC2* 3'UTR region. cDNA was generated from BdTR3C after 3 days of cold in short days. Genomic DNA control and non-templated control are used. 35 cycles were used for PCR.

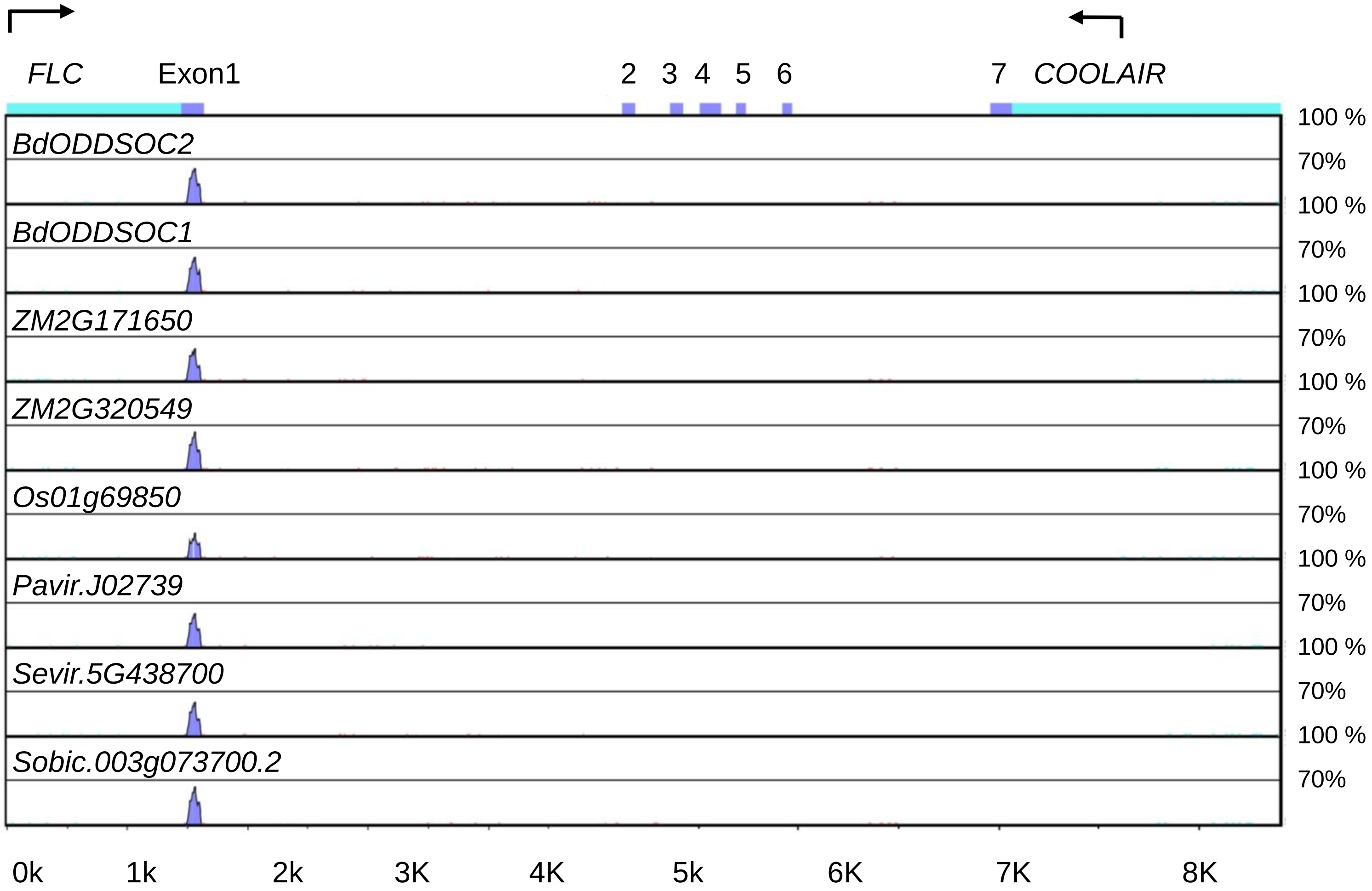

FIGURE S2. The *FLC* homologs in monocots are only similar to *FLC* in the first exon. There is no conserved region with *COOLAIR*. *FLC* gene structure (top) is shown with UTR (light blue) and exons (dark blue). *FLC* homolog genomic sequences are aligned to *FLC* with mVISTA.

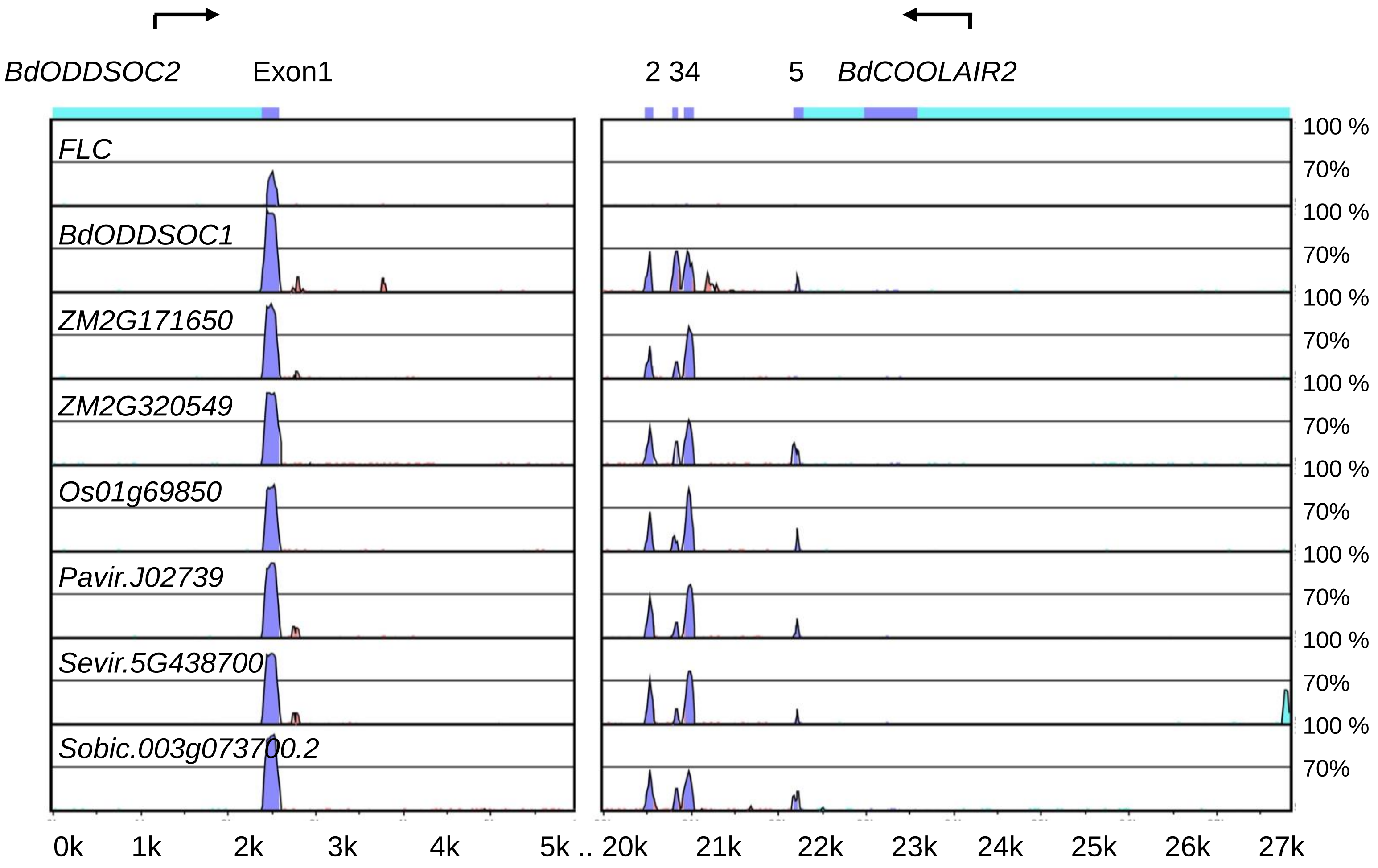

FIGURE S3. There is no conserved region between *BdCOOLAIR2* and lncRNAs annotated for other *FLC* homologs. *FLC* is conserved with *BdODDSOC2* only in the first exon, while *FLC* homologs in monocots are conserved with *BdODDSOC2* in the all five exons. *BdODDSOC2* gene structure (top) is shown with UTR (light blue) and exons (dark blue), *BdCOOLAIR2* is also shown (dark blue). *FLC* and all the other *FLC* homologs genomic sequences are aligned to *BdOODSOC2* with mVISTA.

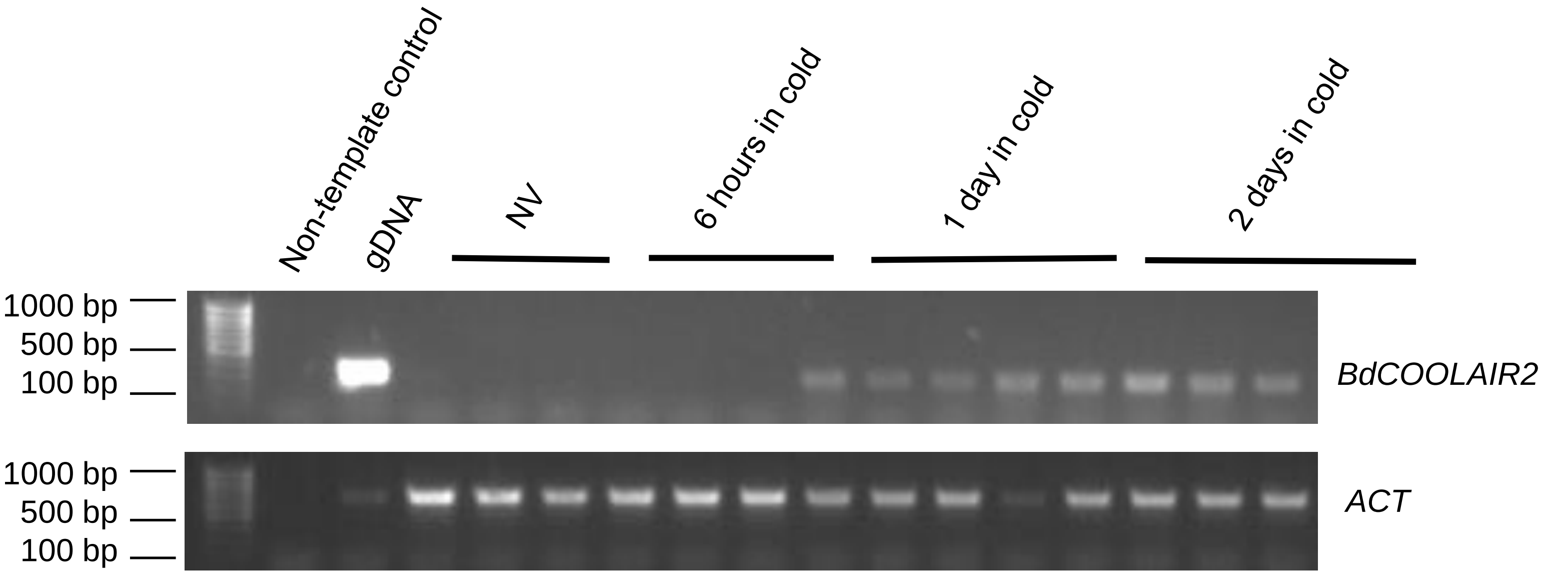

FIGURE S4. Semi-quantitative RT-PCR shows that *BdCOOLAIR2* is induced by cold. RT-PCR with 35 cycles were performed for *BdCOOLAIR2* (up) and *ACT* (down). Non template control and genomic DNA control are used. cDNA was generated from seedlings of non-vernalization, 6 hours in cold, 1 day in cold and 2 days in cold, respectively.

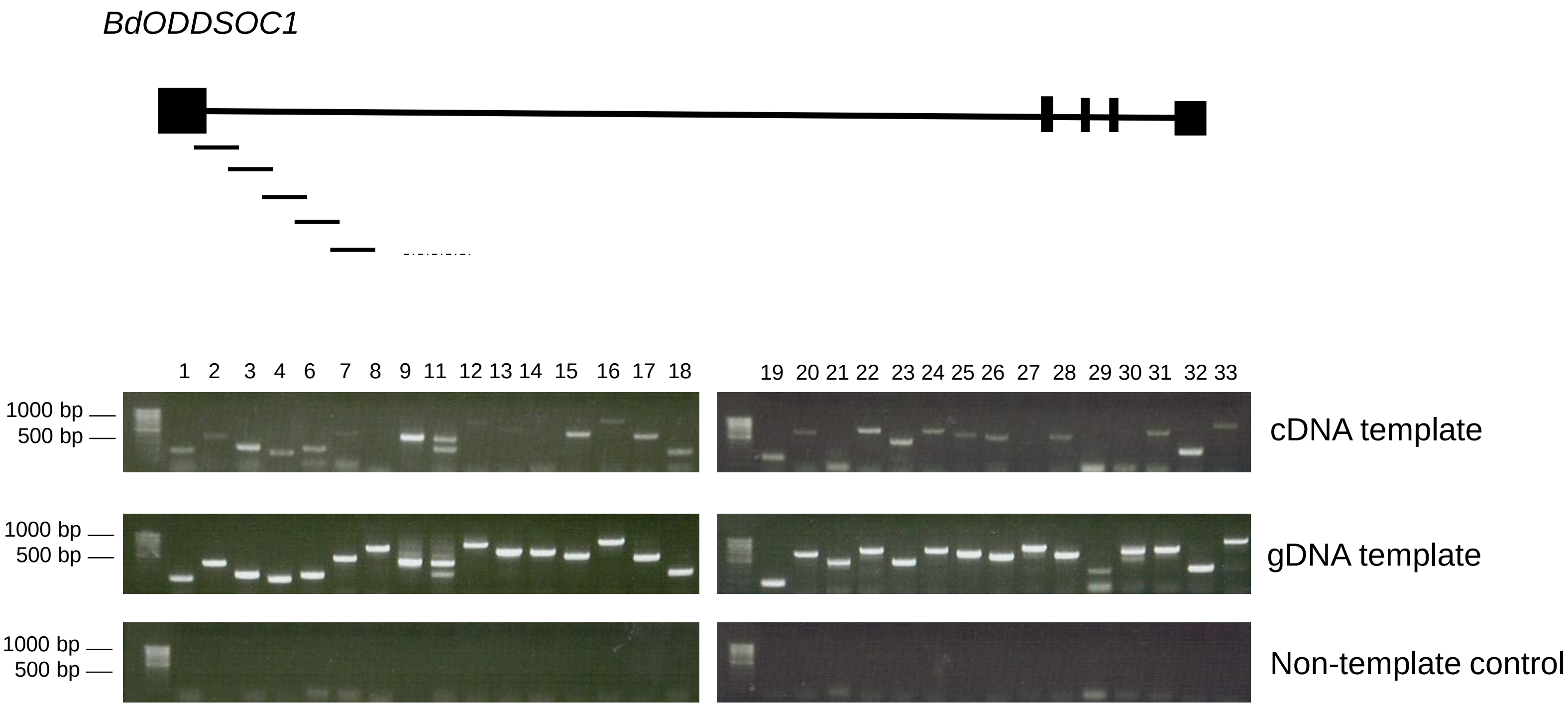

FIGURE S5. RT-PCR of *BdODDSOC1* intronic transcripts. Gene structure of *BdODDSOC1* and primer locations are shown on top. Black boxes indicate exons, long black line indicates introns. Short lines indicate primer spanning regions. Primers pairs for RT-PCR were combined as primer(n)_F + primer(n+1)_R shown in table S3. For example, for intronic region 1, we used primer 1_F and primer 2_R, and so on. cDNA was generated from BdTR3C after 3 days of cold in short days. Genomic DNA control and non-templated control are used. 35 cycles were used for PCR.

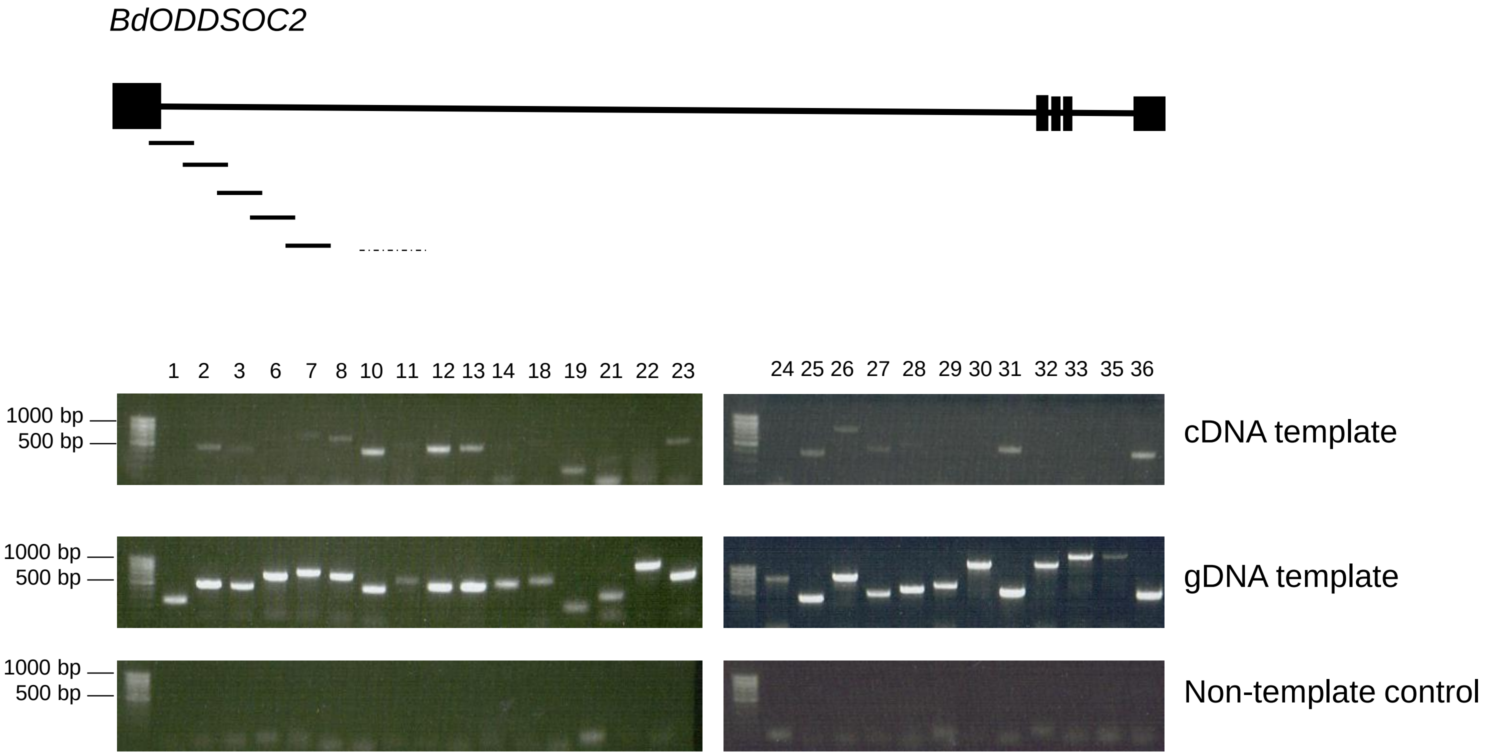

FIGURE S6. RT-PCR of *BdODDSOC2* intronic transcripts. Gene structure of *BdODDSOC2* and primer locations are shown on top. Black boxes indicate exons, long black line indicates introns. Short lines indicate primer spanning regions. Primers pairs for RT-PCR were combined as primer(n)_F + primer(n+1)_R shown in table S2. For example, for intronic region 1, we used primer 1_F and primer 2_R, and so on. cDNA was generated from BdTR3C after 3 days of cold in short days. Genomic DNA control and non-templated control are used. 35 cycles were used for PCR.

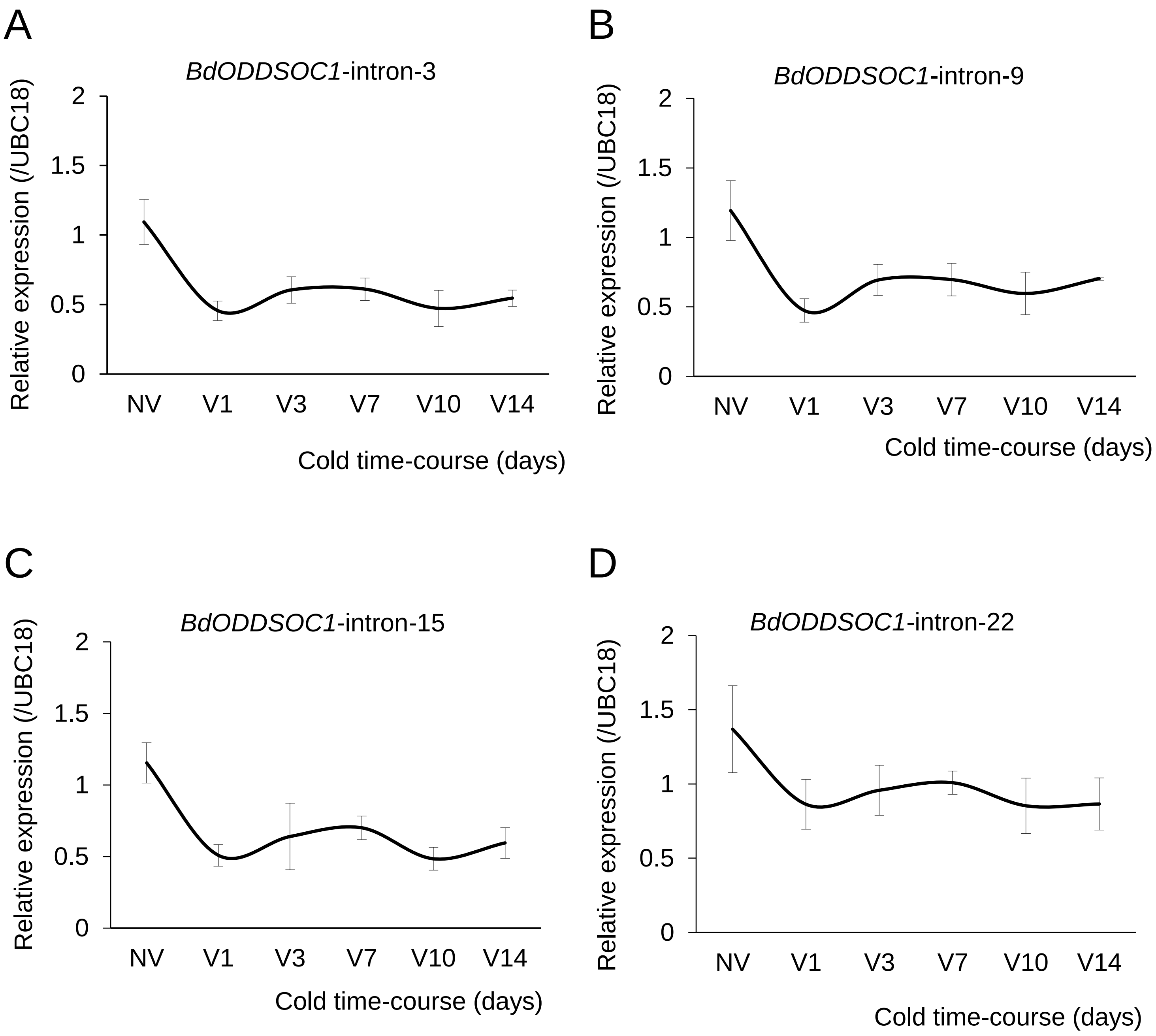

FIGURE S7. Expression of *BdODDSOC1* intronic transcripts. qRT-PCR were performed for 4 different intronic regions of *BdODDSOC1*. NV indicates non vernalization for 3 weeks in short days, V1 indicates 1 day vernalization, and so on. Values are means ± SEM of three biological replicates, two technical replicates. qPCR primer pairs are shown in Supplementary Table S2.

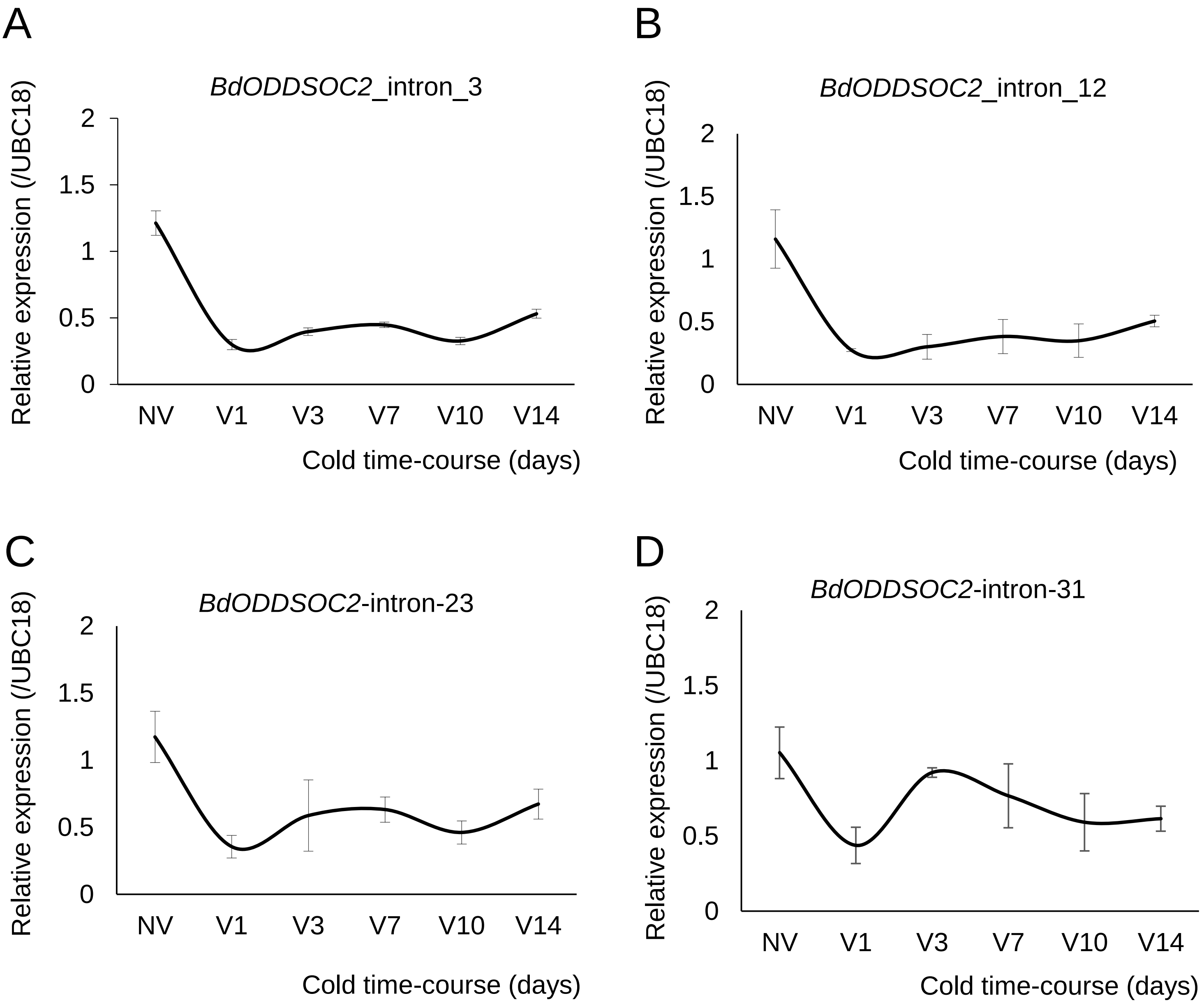

FIGURE S8. Expression of *BdODDSOC2* intronic transcripts. qRT-PCR were performed for 4 different intronic regions of *BdODDSOC2*. NV indicates non vernalization for 3 weeks in short days, V1 indicates 1 day vernalization, and so on. Values are means ± SEM of three biological replicates, two technical replicates. qPCR primer pairs are shown in Supplementary Table S3.

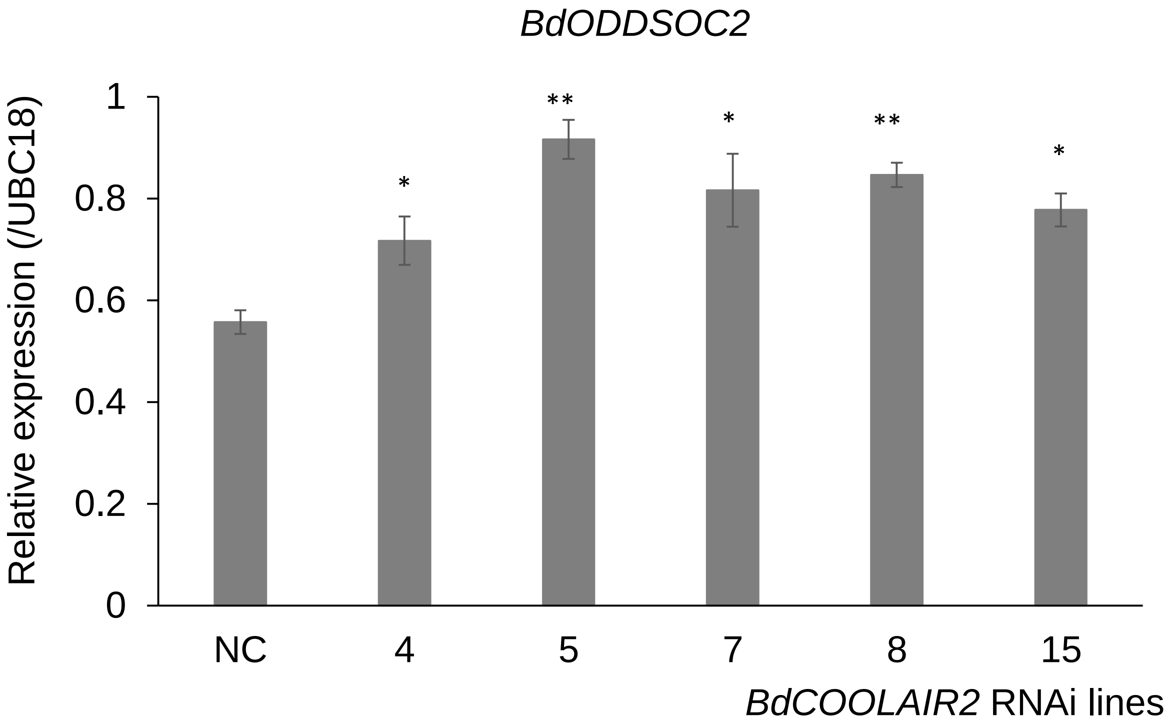

FIGURE S9. Expression of *BdODDSOC2* in accession BdTR3C is higher in *BdCOOLAIR2* RNAi lines than in null sibling controls (NC) after 3 days of vernalization treatment. qRT-PCR were performed for *BdODDSOC2* after 3 days of vernalization treatment. Values are means ± SEM of three biological replicates, two technical replicates. Asterisks indicate statistically significant differences with the student's t-test (*p <0.05, **p <0.01).

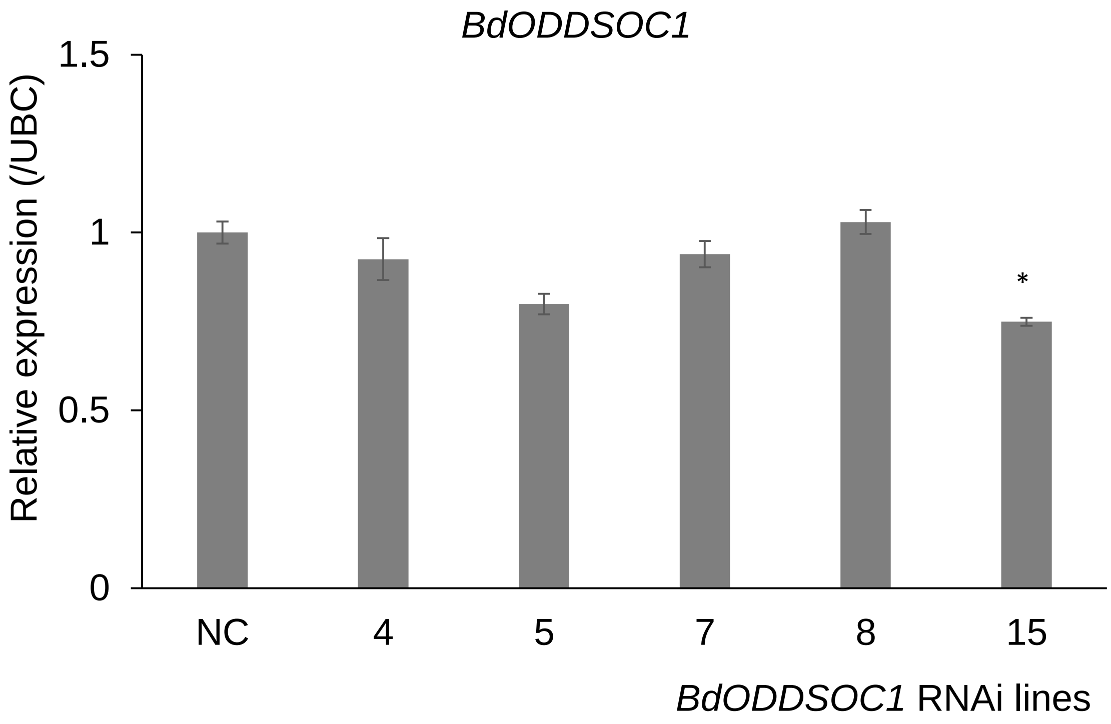

FIGURE S10. Expression of *BdODDSOC1* in accession BdTR3C is not induced in *BdCOOLAIR2* RNAi lines after 3 days of vernalization treatment. Null sibling lines (NC) are used as control**.** qRT-PCR were performed for *BdODDSOC1* after 3 days of vernalization treatment. The expression of *BdODDSOC1* in line 15 is significantly lower than in NC. There is no significant difference of *BdODDSOC1* expression in all the other lines compared with NC. Values are means ± SEM of three biological replicates, two technical replicates. Asterisks indicate statistically significant differences with the student's t-test (*p <0.05).

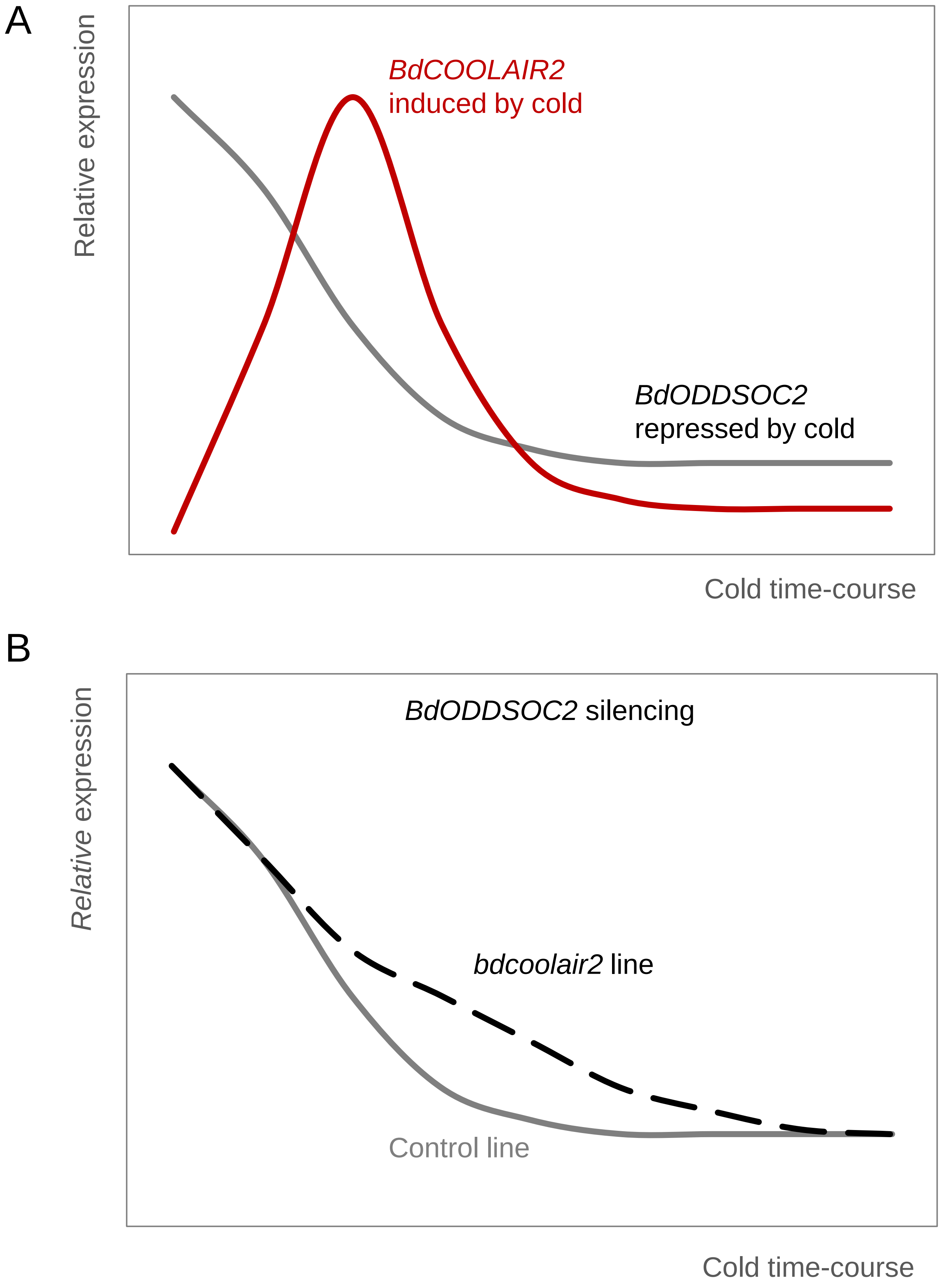

FIGURE S11. *BdCOOLAIR2* function in the silencing of *BdODDSOC2*. (A) *BdODDSOC2* and *BdCOOLAIR2* show opposite expression patterns in response to early vernalization. *BdODDSOC2* goes down (gray) while *BdCOOLAIR2* goes up (red). **(B)** The silencing of *BdODDSOC2* in *BdCOOLAIR2* RNAi lines (black) is slower than in null sibling control lines (gray).

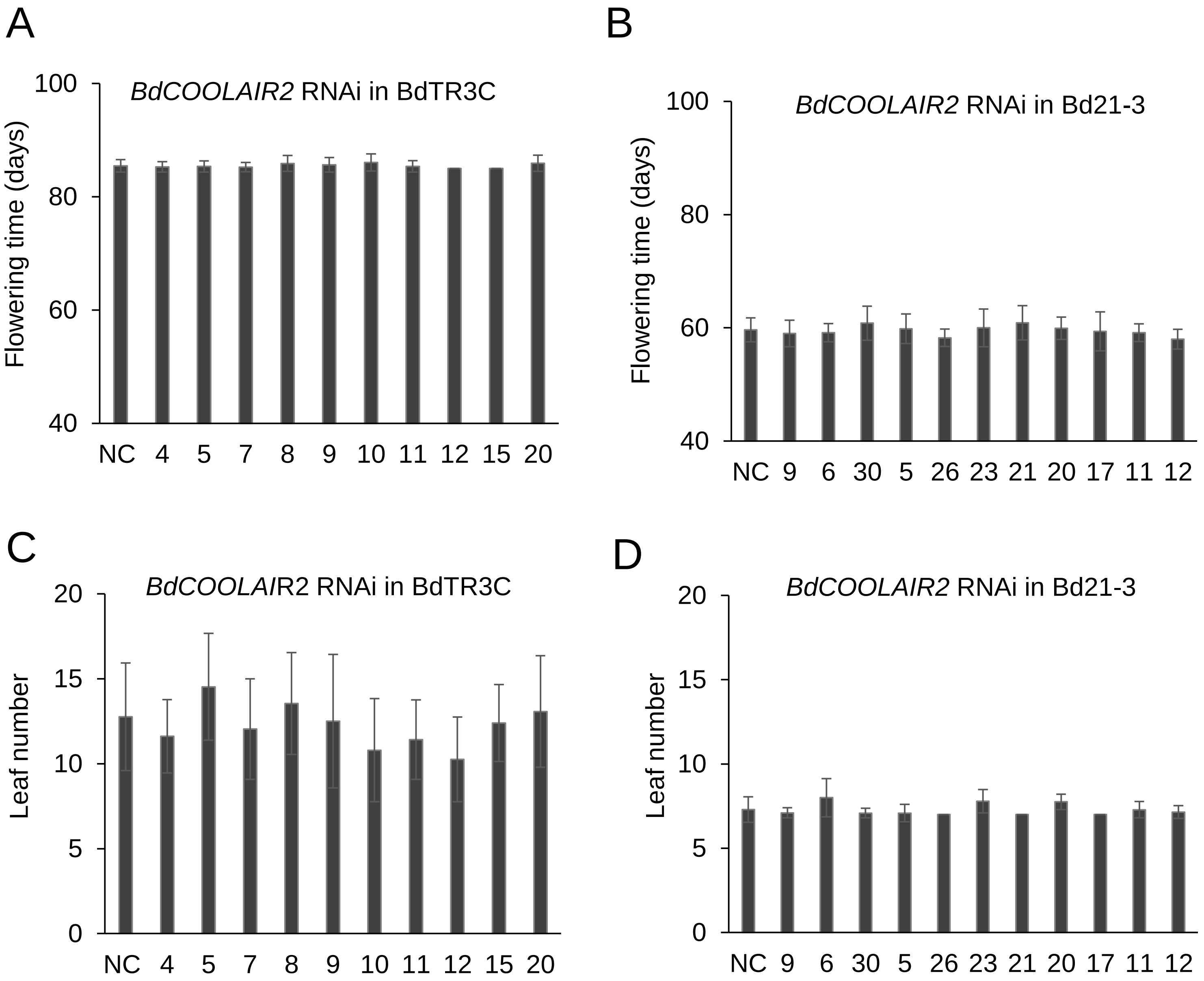

FIGURE S12. *BdCOOLAIR2* knocked down does not affect flowering time and leaf number. RNAi lines were grown in short days for 3 weeks, and treated with 6 weeks vernalization for BdTR3C, 2 weeks vernalization for Bd21-3. Plants were then transferred to long days (20h/4h light/dark) until flowering. NC null sibling control. WT wild type. Values are means ± SD of 8 to 24 biological replicates.

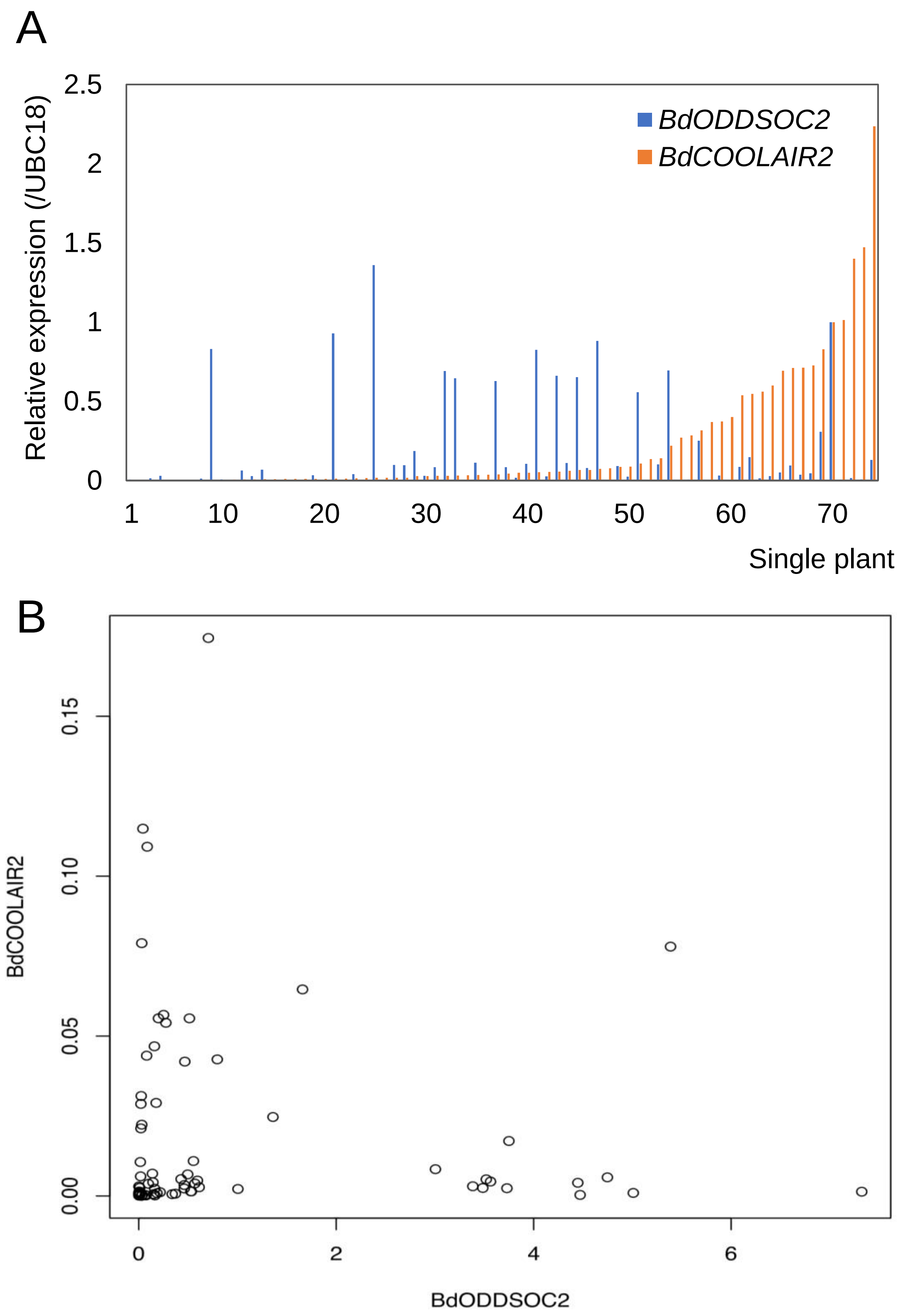

FIGURE S13. Antagonistic expression of *BdODDSOC2* and *BdCOOLAIR2*. qRT-PCR was performed in 74 three-week old plants belonging to different accessions. (A) Values are relative expression (deltadelta Ct) of *BdODDSOC2* (blue) and *BdCOOLAIR2* (orange) in individual plants and expression is normalized to UBC18 and to expression levels in a plant of accession Arn1-1. (B) Plot shows a non co-existence relationship between *BdCOOLAIR2* (y axis) and *BdODDSOC2* (x axis). Values are relative expression normalized to UBC18 (delta Ct).

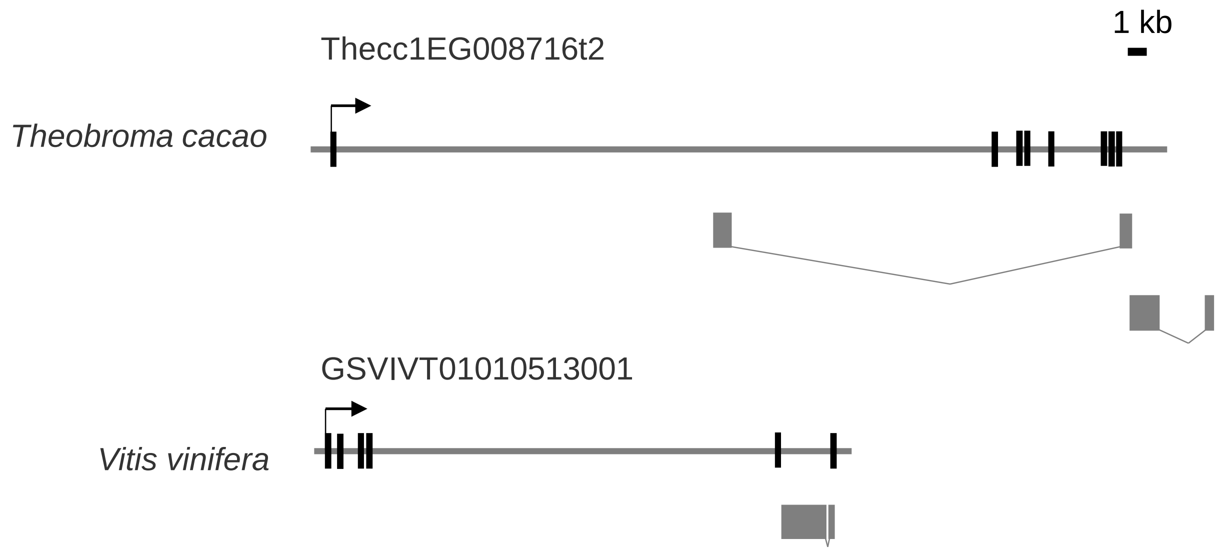

FIGURE S14. Antisense of *FLC homologs* in *Theobroma cacao* and *Vitis vinifera*. Black boxes indicate exons, black lines indicate introns and flanking regions. The direction of sense transcripts (black arrow) is shown.
